# How Does HTLV-1 Undergo Oncogene-Dependent Replication Despite a Strong Immune Response?

**DOI:** 10.3389/fmicb.2017.02684

**Published:** 2018-01-15

**Authors:** Hélène Gazon, Pradeep Chauhan, Malik Hamaidia, Clotilde Hoyos, Lin Li, Roghaiyeh Safari, Luc Willems

**Affiliations:** ^1^National Fund for Scientific Research, Molecular and Cellular Epigenetics, Interdisciplinary Cluster for Applied Genoproteomics, Liège, Belgium; ^2^Molecular Biology, TERRA, Gemboux Agro-Bio Tech, Gembloux, Belgium

**Keywords:** HTLV, BLV, microRNA, cytotoxic T cells, long non-coding RNA, Leukemia

## Abstract

In 1987, Mitsuaki Yoshida proposed the following model (Yoshida and Seiki, 1987): “... T-cells activated through the endogenous p40x would express viral antigens including the envelope glycoproteins which are exposed on the cell surface. These glycoproteins are targets of host immune surveillance, as is evidenced by the cytotoxic effects of anti-envelope antibodies or patient sera. Eventually all cells expressing the viral antigens, that is, all cells driven by the p40x would be rejected by the host. Only those cells that did not express the viral antigens would survive. Later, these antigen-negative infected cells would begin again to express viral antigens, including p40x, thus entering into the second cycle of cell propagation. These cycles would be repeated in so-called healthy virus carriers for 20 or 30 years or longer....” Three decades later, accumulated experimental facts particularly on intermittent viral transcription and regulation by the host immune response appear to prove that Yoshida was right. This Hypothesis and Theory summarizes the evidences that support this paradigm.

## Introduction

At least 20 million people worldwide are infected with human T-cell leukemia virus type 1 (HTLV-1) (Gessain and Cassar, 2012; Bangham, 2017; Watanabe, 2017). This retrovirus is prevalent in southwestern Japan, sub-Saharan Africa, the Caribbean islands, South America, the Middle East and Austro-Melanesia. Transmission occurs principally from mother to child via milk or between sexual partners through contaminated blood. Infected individuals are at risk of developing a rapidly progressive malignancy, adult T-cell leukemia/lymphoma (ATLL), and a debilitating neurologic condition, HTLV-1 associated myelopathy/tropical spastic paraparesis (HAM/TSP) (Willems et al., 2017).

Although frequently neglected in the field, bovine leukemia virus (BLV) is a useful model to address specific questions that cannot be answered in the HTLV-1 system (Rodríguez et al., 2011; Polat et al., 2017). Both viruses are indeed closely related δ-retroviruses that induce hematological diseases. In the bovine species, the most prevalent clinical manifestation in about one-third of infected animals is persistent lymphocytosis, a benign accumulation of infected B-lymphocytes (Gutierrez et al., 2014). In a minority of cases (about 5–10%), BLV infection can progress to fatal leukemia/lymphoma whose most dramatic consequence is spleen hypertrophy and disruption consecutive to tumor formation. BLV typically persists in less than 1% of peripheral blood cells, leading to an asymptomatic infection in the majority of infected animals. BLV is transmitted horizontally by direct contact, iatrogenic procedures or insect bites upon transfer of infected cells from milk, blood, and body fluids from heavily infected dams (Barez et al., 2015).

## The Paradox: Htlv-1 Replication is Driven by Oncogenic Proteins that Expose the Infected Cell to the Host Immune Response

According to currently most accepted model, two viral proteins (Tax and HBZ, HTLV-1 bZIP) are hypothesized to have the highest impact on viral replication and cell transformation (Matsuoka and Jeang, 2007; Carpentier et al., 2015). The modes of action of Tax and HBZ are remarkably pleiotropic and involve a variety of cell signaling pathways (CREB, NF-κB, AKT, and TNF) (Twizere et al., 2003; Boxus et al., 2008). Tax inhibits tumor suppressors (p53, Bcl11B, and TP53INP1) and activates cyclin-dependent kinases (CDKs), both of these mechanisms leading to accelerated cell proliferation. Experimental evidence further shows that Tax drives tumor formation in transgenic mouse models, supporting its oncogenic potential. Through an interaction with the helicase complex (mcm2-7), Tax accelerates S phase progression by initiating additional replication origins. By promoting unscheduled cell growth, Tax also induces genomic instability and generates somatic alterations. Another viral protein, HBZ also favors cell proliferation by inhibiting apoptosis/senescence and modulating the cell cycle (Ma et al., 2016). In fact, the HBZ protein counteracts a series of Tax-activated viral and cellular pathways (such as NF-κB, Akt, and CREB). Transgenic expression of HBZ in CD4 + T cells induced T-cell lymphomas and systemic inflammation in mice, resembling diseases observed in HTLV-1 infected individuals (Satou et al., 2011).

A major issue of expression of viral proteins is initiation of the host immune response. Indeed, Tax induces a strong immune response that would be harmful to infected cells (Nagai et al., 2001; Kannagi et al., 2005; Rowan et al., 2014). In comparison, HBZ triggers a less efficient immunity that is consistent with low expression low expression throughout HTLV-1 infection (MacNamara et al., 2010). How does HTLV-1 persist despite a strong immune response against viral oncogenes that promote infected cell replication? The next paragraph lists the experimental evidence pertaining to this paradox.

## Experimental Evidence and Interpretations

### Evidence 1: Infected Cells Proliferate Faster to Undergo Clonal Expansion

The BLV model has been instrumental to quantify the dynamics of cell turnover *in vivo* (Florins et al., 2007). Experiments based on intravenous injection of bromodeoxyuridine (BrdU) and carboxyfluorescein diacetate succinimidyl ester (CFSE) demonstrate that B-lymphocytes are proliferating significantly faster in BLV-infected sheep than in healthy controls (Debacq et al., 2002, 2006). Excess of proliferation is compensated by an increase in cell death, thereby maintaining homeostasis. Increased cell proliferation is also reported in HTLV-induced HAM/TSP using a similar strategy based on incorporation of deuterated glucose (Asquith et al., 2007). *In vivo*, BLV and HTLV infection is thus characterized by an increased cell turnover, likely driven by viral oncogenes such as Tax and HBZ.

### Evidence 2: 5′ LTR Directed Transcription Is Extremely Low *in Situ*

The amount of viral RNA transcribed from the 5′ LTR promoter is extremely low *in vivo*. In primary tumor cells, only very sensitive techniques such as RT-PCR can identify viral RNA transcribed from the 5′ LTR promoter (Lagarias and Radke, 1989; Rovnak and Casey, 1999; Shimizu-Kohno et al., 2011; Demontis et al., 2015). It was initially concluded that the provirus is silent. However, *in situ* experiments showed that rare cells expressed large amounts of viral transcripts (Lagarias and Radke, 1989).

### Evidence 3: Sense Transcription Can Be Activated by Various Stimuli

The main regulatory element of the 5′ LTR promoter that is activated by Tax is a 21 bp enhancer that interacts with CREB/ATF transcription factors (Suzuki et al., 1993; Adam et al., 1996). This complex activates sense transcription when cells are isolated *ex vivo* from HTLV-1 carriers or BLV-infected sheep. This reactivation can further be increased by various stimuli such as polyclonal activators, HDAC inhibitors or oxygen deprivation (Kerkhofs et al., 1996; Achachi et al., 2005; Tajima and Aida, 2005; Lezin et al., 2007; Olindo et al., 2011; Kulkarni et al., 2017).

### Evidence 4: Immunity against Most Viral Antigens Is Extremely Efficient While HBZ Is Poorly Immunogenic

Persistent infection by BLV and HTLV-1 is characterized by a permanent and vigorous immunity against viral antigens (Kannagi et al., 2005; Burbelo et al., 2008; Bangham et al., 2009; Kattan et al., 2009). This immune response efficiently controls viral replication *in vivo* as demonstrated in the BLV model (Florins et al., 2006, 2009, 2011; Gillet et al., 2013). Among viral proteins, Tax is the immunodominant HTLV-1 antigen in the T-cell response (Kannagi et al., 1991; Goon et al., 2004). In contrast, the HBZ protein is very poorly immunogenic and expressed at very low levels in infected cells (Suemori et al., 2009; Enose-Akahata et al., 2013; Rowan et al., 2014; Raval et al., 2015; Baratella et al., 2017). Humoral immunity against HBZ protein is indeed particularly weak (Raval et al., 2015; Shiohama et al., 2016). However, cytotoxic T cells specific to HBZ but not to the immunodominant Tax are the most effective in the control of HTLV-1 (MacNamara et al., 2010). These results thus focus attention on the extremely low expression and low immunogenicity of HBZ.

### Evidence 5: Antisense Transcripts and microRNAs Are Abundantly and Permanently Expressed in Tumors

In contrast to 5′ LTR directed transcription, antisense RNA synthesis initiating at the 3′ LTR is consistently identified in primary ATL cells and BLV tumors (Usui et al., 2008; Saito et al., 2009; Durkin et al., 2016). In contrast to HTLV-1, BLV also abundantly transcribes a cluster of microRNAs from internal pol III promoters (Kincaid et al., 2012; Rosewick et al., 2013; Van Driessche et al., 2016). These microRNAs are required for efficient viral replication and induction of pathogenesis (Gillet et al., 2016).

### Evidence 6: HBZ and AS RNAs are Mainly Localized in the Nucleus Suggesting a Role in Epigenetics

The HBZ RNA is mainly localized in the nucleus, consistent with a low rate of translation (Hivin et al., 2005; Rende et al., 2011; Shimizu-Kohno et al., 2011; Li et al., 2012; Raval et al., 2015). Although the function of the HBZ protein has been clearly evidenced, the dominant nuclear localization of the HBZ RNA thus suggests other regulatory roles such as for example epigenetic modulation of gene expression. In BLV, the antisense transcripts are not predicted to be translated but are rather primarily retained in the nucleus, hinting at a lncRNA-like role (Durkin et al., 2016).

### Evidence 7: Untranslated HBZ RNA Has a Function

The oncogenic role of the HBZ RNA was revealed by an untranslatable HBZ RNA (i.e., devoid of initiation codon) able to induce the proliferation of a human IL-2-dependent T-cell line (Kit225) (Satou et al., 2006). Microarray expression analysis reveals that HBZ RNA and protein differentially modulate the transcription of host genes. HBZ RNA activates the transcription of survivin and cell-cycle related genes (Mitobe et al., 2015). Thus, the HBZ gene has bimodal functions in two different molecular forms as a polypeptide and a ribonucleic acid. Notwithstanding important activities as protein, the scarcity of the HBZ polypeptide in tumor cells contrasts with the expression of the HBZ RNA. Therefore, main functions of HBZ are exerted by its RNA form. Although the level of expression of HBZ protein is unquestionably very low, the protein must nevertheless play an essential role in the life-cycle of the virus because the coding sequence has been conserved during evolution in presence of a protective host immune response.

### Evidence 8: Alternate Transcription of Sense and Antisense Transcription

Single cell analysis shows that the Tax RNA is expressed in bursts and is exported from the nucleus, whereas the majority of hbz RNA is retained (Billman et al., 2017). Time-lapse imaging of destabilized enhanced green fluorescent protein indicates that Tax expression is transient, fluctuates between on/off states and is detected only in HBZ-negative cells (Jasunaga and Matsuoka, 2017; Mahgoub et al., 2017). Viral persistence is thus characterized by successive cycles of sense/antisense transcription. Evidence from single-molecular RNA-FISH nevertheless indicates a more complex relationship between expression of the two strands that are not transcribed in strict alternation (Billman et al., 2017).

## A Model for Viral Persistence Under Immune Control

**FIGURE 1:**
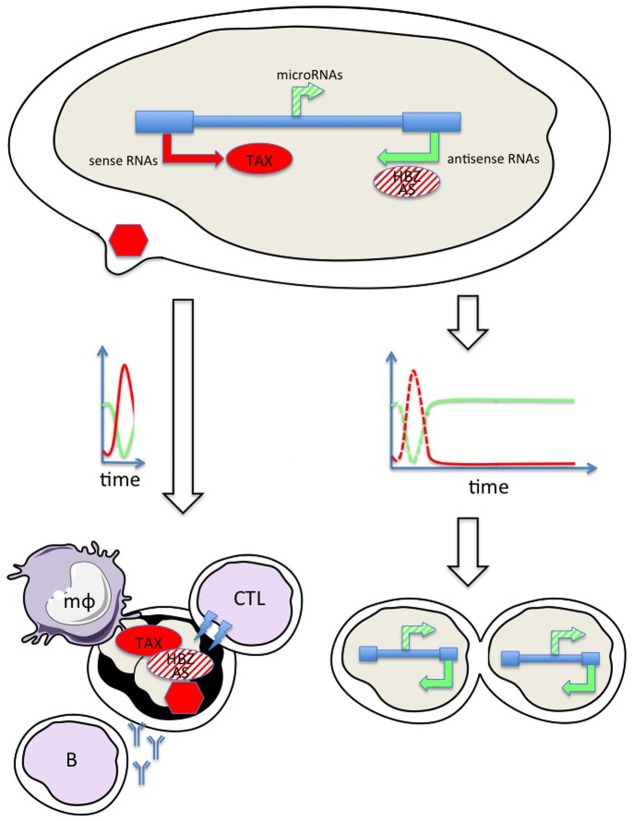
Hypothetical model combining temporal regulation of viral expression allowing escape from host immunity. Sense transcription (red arrow) directed by the 5′ LTR promoter allows translation of the TAX protein (red ellipse) and synthesis of the viral particle (red hexagon). The antisense strand is transcribed in a HBZ (HTLV-1) and (AS) BLV RNA (green arrow) and protein (ellipse hatched in red). BLV, but not HTLV, also encodes RNA polymerase III driven microRNAs (hatched green arrow). Burst of sense transcription (red line on the time graph at the left) transiently exposes infected cells to the immune response: cytotoxic T cells (CTL), antibodies from B-lymphocytes (B) and macrophages (mϕ). Silencing of sense transcription, for example by initiating antisense RNA synthesis (graph on the right) allows further cell proliferation.

These experimental evidences are consistent with the model presented in **Figure 1** and support Yoshida’s paradigm.

BLV/HTLV proviruses are transcribed in both orientations. Sense transcription from the 5′ LTR (red arrow) generates genomic and spliced subgenomic RNAs (e.g., Tax). Translation of these RNAs into oncogenic proteins (Tax) triggers cell proliferation, mitosis, and clonal expansion of the infected cell. Synthesis of structural (gag, env) and enzymatic (protease, reverse transcriptase, and integrase) proteins are required for assembly of the viral particle (red hexagon) that will further colonize new target cells. The provirus is also transcribed in the antisense orientation from the 3′ LTR (green arrow) yielding HBZ and AS RNAs for HTLV and BLV, respectively. HBZ is very poorly translated while the coding potential of AS is ambiguous (hatched in red). Furthermore, the immunogenicity of HBZ is weak compared to all other viral antigens. BLV, but not HTLV, also encodes RNA polymerase III driven microRNAs (hatched green arrow). RNA synthesis from the 5′ LTR is mostly silent but burst of sense transcription (red line on the time graph) transiently exposes infected cells to the immune response (e.g., cytotoxic, humoral, and innate). The only option that allows survival is to silence sense transcription, for example by initiating antisense RNA synthesis (graph on the right). This simplified model is nevertheless incomplete because the infected cell is also exposed to the CTL response to HBZ providing that the protein is expressed. Experimental evidence indicates that the HBZ RNA is mostly nuclear and is therefore not translated. Single cell kinetics of RNA and protein expression would answer to this still unsolved question. Another issue is the role of the intrinsic immunity operating within virus-infected cells, i.e., restriction factors (RFs) inhibiting Tax function or reverse transcription of viral genome (Tosi et al., 2011; Bai and Nicot, 2015; Forlani et al., 2016).

## Conclusion

Yoshida predicted that the Tax oncogene should be expressed in cycles to allow cell survival. Recent reports describing bursts between sense and antisense transcriptions are consistent with this hypothesis. Regular switches between 5′ and 3′ transcription indeed allows transient Tax expression and fast silencing of viral expression. This mechanism would allow Tax-driven cell proliferation and synthesis of viral particles in presence of the host immunity. This model thus illustrates the dynamic equilibrium between a virus attempting to proliferate under a tight control exerted by the immune response.

## Author Contributions

All authors have contributed equally to this work. LW drafted the manuscript. All authors corrected, edited, and approved the paper.

## Conflict of Interest Statement

The authors declare that the research was conducted in the absence of any commercial or financial relationships that could be construed as a potential conflict of interest.
